# The Role of Platinum-Based Chemotherapy in the Pathogenesis of Tendinosis in Cancer Patients

**DOI:** 10.7759/cureus.87791

**Published:** 2025-07-12

**Authors:** Joseph Bisiani, Brian Lynch

**Affiliations:** 1 Department of Orthopedics, Renaissance School of Medicine, Stony Brook University, Stony Brook, USA

**Keywords:** cancer patients, carboplatin, cisplatin, collagen, oxaliplatin, platinum-based chemotherapy, tendinosis, toxicity

## Abstract

Currently, there are numerous anti-cancer drugs to prevent the spread of different neoplasms. Patients who depend on these medications as a form of treatment look to hopefully minimize the spread of cancer. However, there are well-known side effects of platinum-based chemotherapies, such as different electrolyte imbalances. These disturbances could possibly lead to the pathogenesis of tendinosis among cancer patients who take long-term platinum-based chemotherapy treatments, something that has not been thoroughly studied and warrants further investigation. Electrolyte disturbances in tendons can lead to dehydration, which can impair collagen synthesis and ultimately lead to tendon degeneration among cancer patients who are utilizing these long-term platinum cancer drugs.

## Introduction and background

Introduction: history, use, and side effects of platinum-based chemotherapies

Platinum-based anti-cancer drugs have been in use for many years now. They were first developed with the intention to inhibit cell division. Many cancer patients rely on platinum-based drugs for chemotherapy, as these medications are able to cause DNA damage and induce apoptosis of cancer cells [1]. A well-known side effect of platinum-based chemotherapies, including cisplatin and carboplatin, is electrolyte imbalances, including hypomagnesemia, hypokalemia, hypophosphatemia, hypocalcemia, and hyponatremia [2]. Cisplatin, one of the most common platinum-based analogs, was discovered back in 1845, but its biological properties were not uncovered until 1965, when Barnett Rosenberg explored its mechanism of inhibiting cell division [3]. These drugs have been in use for decades, and their use has grown for the treatment of numerous types of cancers. Cisplatin has a therapeutic effect on many malignant tumors, including breast, ovarian, and colorectal cancers [4]. Platinum-based analogs can be used for a wide range of tumors, and this makes them very diverse in their applications.

Anti-cancer drugs are typically prescribed for fairly long periods of time to exert their full effects of inhibiting the specified cancer. For instance, for platinum-based chemotherapy, most guidelines typically recommend a maximum of four to six cycles, with four chemotherapy cycles most commonly being recommended as the optimal regimen [5]. Each cycle of these chemotherapies typically lasts three to four weeks. If you calculate the total duration that cancer patients typically take these platinum-based analogs, the total time on the therapy can range from four months to half a year. During the course of treatment, it is plausible that the side effects of these drugs could arise and that drug toxicity can occur.

The well-studied side effects of platinum-based chemotherapies include a wide range of symptoms; for instance, the dose-limiting side effect for cisplatin is nephrotoxicity, for carboplatin it is myelosuppression, and for oxaliplatin it is neurotoxicity. Other common side effects include anaphylaxis, cytopenias, hepatotoxicity, ototoxicity, cardiotoxicity, nausea, vomiting, and diarrhea [6]. One of the unique side effects of drugs such as cisplatin or carboplatin is their contributions to different electrolyte disturbances. Platinum compounds are associated with inducing lower levels of sodium, potassium, and magnesium [7]. These side effects of electrolyte disturbances may pose a risk for tendons, as tendons rely on normal electrolyte values to maintain proper collagen levels. If a cancer patient develops low electrolyte levels due to long-term platinum-based antineoplasm therapy, it may impair collagen development, which can lead to tendinosis and subsequently tendon deterioration. This may have drastic effects on patients’ normal range of motion and function. Therefore, the use of platinum-based anti-cancer drugs could play a role in the pathogenesis of tendinosis in cancer patients who rely on these pharmacological agents long-term, affecting their mobility and ability to perform daily activities.

## Review

Mechanism of action of platinum-based anti-cancer drugs

**Figure 1:**
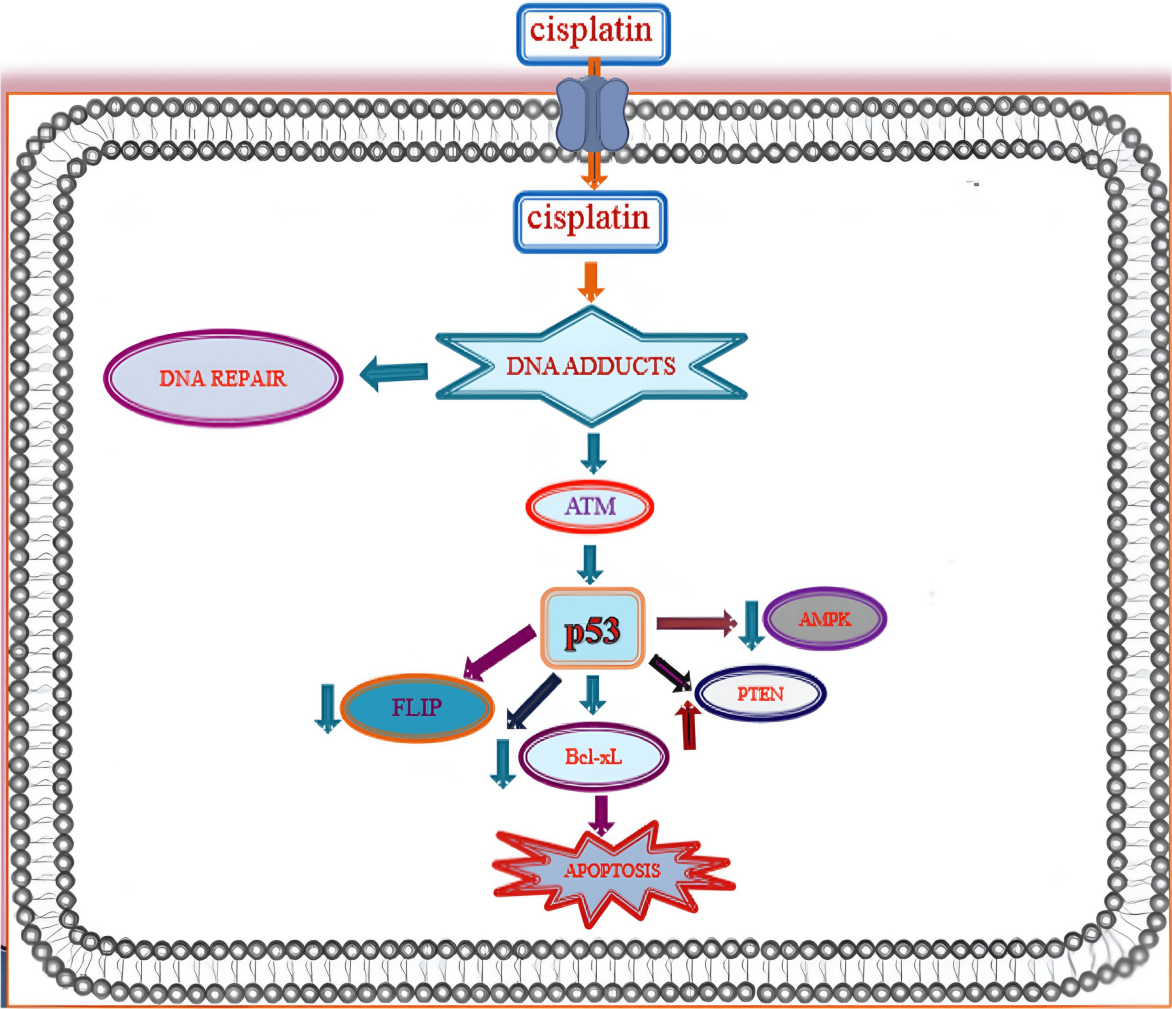
Overview of molecular mechanisms of cisplatin cytotoxicity [9]

Platinum-based analogs for cancer treatment have a unique mechanism of action in preventing the spread of different cancers. Specifically, the mechanism for cisplatin involves non-cell cycle-specific cytotoxicity, which is accomplished via covalent binding of platinum to the purine bases guanine and adenine. Through this covalent binding, intra-strand and inter-strand crosslinks form, which cause subsequent strand breaks in DNA [8]. Through this unique mechanism, platinum-based chemotherapies target rapidly dividing cells with their ability to break DNA, all of which can help stop very malignant neoplasms. Figure 1 depicts the unique mechanism by which cisplatin (a very common platinum-based chemotherapy) is able to promote apoptosis [9].

The unique mechanism of cisplatin is how it enters cancer cells and interacts with DNA to form DNA adducts. It also regulates protein kinase and activates p53, which leads to a series of signaling cascades and overall apoptosis in cancer cells. Platinum-based analogs for cancer treatment often help stop malignant cancers through the formation of interstrand crosslinks that damage the DNA needed for cell replication. When cancer cells are unable to adequately replicate due to damaged DNA, metastasis is hindered and cancer inhibition occurs.

Renal damage and electrolyte disturbances

Cisplatin (in addition to the other platinum-based anti-cancer agents) is a coordinate metal complex that poses severe effects on the renal system, including both acute and chronic renal insufficiency. Nephrotoxicity may occur in as many as 50% to 75% of patients receiving this therapy and is due to renal tubular injury [10]. The mechanism by which platinum-based drugs do this is by reducing the glomerular filtration rate and causing severe renal tubular damage [11]. In particular, the proximal tubule is known for reabsorption of very important cations and anions needed for proper electrolyte maintenance. The proximal tubule is where the majority of sodium reabsorption takes place [12]. If there is damage to the proximal tubule, sodium wasting and hyponatremia can occur.

The proximal tubules are exposed to cisplatin during its biphasic excretion into the urine, with the highest levels of exposure arising during the first three hours after administration [13]. The toxicity is dose- and time-dependent, and damage to the proximal tubules is first observed three to four days after the administration of cisplatin. When this occurs, it results in electrolyte imbalances with hyponatremia and low electrolyte values in cancer patients taking long-term platinum-based analog anti-cancer therapies.

Hyponatremia is a severe issue that can cause a wide range of symptoms among patients. To start, water and sodium follow each other very closely, and low levels of sodium due to sodium excretion in the urine can lead to high natriuresis due to water following the sodium. This can lead to a low volume state, also known as hypovolemia. In addition, drugs such as cisplatin can cause low electrolyte values. Studies looking at magnesium levels demonstrated that platinum-based chemotherapies can lead to hypomagnesemia. One study revealed that there was a statistically significant decrease in magnesium levels from baseline to the lowest magnesium level (mean = 0.68 mmol/L, standard deviation = 0.13) (P < 0.0005) during cisplatin-based chemotherapy. The incidence of hypomagnesemia (serum magnesium < 0.7 mmol/L) at any point during chemotherapy was 43% [14]. There is clear evidence that platinum-based antitumor drugs do lead to low electrolyte levels, all of which can pose negative effects on different organ systems.

This side effect has been demonstrated in other platinum-based anti-cancer drugs besides cisplatin. From January 2002 to April 2009, a study was conducted on 80 hyperthermic intraperitoneal chemotherapy (HIPC) procedures. Of these 80 patients, 20 (25%) received oxaliplatin (dose range 300 × 400 mg/m^2^) carried in 5% dextrose solution. In patients receiving HIPC with oxaliplatin, electrolyte disturbances were the most common complication. Compared with MMC (mitomycin C, an antitumor antibiotic), patients receiving oxaliplatin had significant 24-hour postoperative uncorrected hyponatremia (P < 0.001), corrected hyponatremia (P < 0.001), hyperglycemia (P < 0.001), and metabolic acidosis (P < 0.001) [15]. Hyponatremia observed in platinum-based cancer treatments can cause severe issues in patients and can even eventually lead to impaired collagen synthesis, inevitable tendinosis, and tendon degeneration.

The effect of low electrolytes on collagen and tendon function

Low electrolyte levels can have negative effects on overall collagen levels and impaired collagen production. Collagen is an essential protein that is involved in the formation of fibrillar and microfibrillar networks of the extracellular matrix, basement membranes, and other structures of the extracellular matrix [16]. It is found in different parts of the body but is especially important for tendon integrity. Tendons are composed of highly ordered extracellular matrix, in which collagen molecules assemble into filamentous collagen fibrils, which then go on to aggregate and form collagen fibers [17]. Because of this, collagen provides immense importance to a tendon’s overall strength and health.

When a person develops low sodium levels, water leaves with the sodium, and high levels of natriuresis can occur, leading to a low volume state. When this occurs, collagen can be severely weakened due to this dehydrated state. This is because a typical tendon consists of 70% of water and 30% of dry mass, which is composed of 60-80% of type I collagen and 2% of elastin [18]. Type 1 collagen relies heavily on hydration for its overall integrity, as it is normally present in a hydrated form [19]. Therefore, if a cancer patient develops hyponatremia due to longstanding use of platinum-based anti-cancer drugs, this excessive sodium excretion can lead to a dehydrated state that can impair overall collagen strength needed for tendon function, leading to tendinosis. Studies have even shown that uncrosslinked collagen membranes in water showed a very high water-absorption capability, clearly visible from the significant swelling ratio (close to 10) and thickness increase [20]. The importance of hydration for type 1 collagen, an important part of tendon integrity, is very high, and in low volume states, collagen structure can be weakened and deteriorate. This collagen deterioration and impaired collagen synthesis can pose negative effects on a tendon and lead to tendinosis over time, as tendons rely on collagen for their overall structure and function. Therefore, when patients take platinum-based anti-cancer drugs for long periods of time, one of the common side effects of electrolyte wasting could plausibly induce collagen deterioration and subsequent tendon weakness.

Tendinosis and effects on cancer patients

Cancer patients who are on long-term platinum-based antitumor therapy can develop impaired collagen synthesis and type 1 collagen deterioration due to a low volume state. This can lead to tendinosis and lessened mobility. Low volume and dehydration impair collagen synthesis, specifically due to their effects on tenocytes. The characteristic cells in tendons and ligaments are called tenocytes, which are responsible for the formation and turnover of the extracellular matrix. Tenocytes react to external stimuli and facilitate the functional adaptation of the proteoglycan and collagen network to mechanical requirements needed for movement of the human body [21]. Hence, if a patient on platinum-based cancer treatments develops electrolyte disturbances, collagen synthesis in tenocytes can be impaired. This can lead to weakened tendon strength and tendinosis.

When tendinosis occurs, the overall strength of the tendon is weakening due to deterioration of the tendon. This can lead to severe effects on patients, as complications with tendinosis can include contractures of the tendon, tendon adhesions, atrophy of muscles, and loss of functionality, even up to and including disability [22]. Therefore, this possible development of tendinosis from long-term use of platinum-based anti-cancer drugs is extremely important to understand and analyze. Cancer patients could develop lessened mobility and muscle atrophy, all of which pose risk for altering their daily activities and range of motion. If platinum-based antitumor drugs play a possible role in the development of tendinosis in cancer patients, treatments, such as adequate hydration [23], to help the renal system or limiting the duration of drug use can help possibly minimize these hypothesized effects.

Tendinosis can have drastic effects on the day-to-day mobility of an individual. Weakening tendon strength can make it hard to move certain muscles, which can lead to alterations in overall movement and success in accomplishing daily tasks. The complexities behind tendinosis treatment can actually further enhance these issues of platinum-based cancer drugs as well. For instance, in the case of nonsurgical treatment for chronic Achilles tendinosis, a combination of rest, non-steroidal anti-inflammatory drugs (NSAIDs), a correction of malalignments, and stretching and strengthening exercises are utilized, but there is sparse scientific evidence supporting the use of most proposed treatment regimens [24]. NSAIDs are known to inhibit prostaglandin synthesis, which inherently leads to afferent arteriole constriction [25]. This can reduce the glomerular filtration rate, which, in turn, can lead to lessened excretion of the platinum anti-cancer drugs, worsening its drug toxicity. This is an important consideration to keep in mind, as certain medications, such as NSAIDs, can have worsening implications on the hypothesized role that platinum-based anti-cancer drugs have in tendinosis pathogenesis in cancer patients.

Immune responses and the role in tendon healing

One important aspect of the role of platinum anti-cancer drugs in tendon health is the effect of these drugs on the immune system, which may actually counteract the aforementioned tendinosis development. These drugs are known to actually promote the immune system. For instance, platinum-based anticancer drugs can enhance the immunostimulatory potential of DCs and decrease the immunosuppressive capacity of tumor cells. By doing this, there is inhibition of STAT6-mediated expression of co-inhibitory molecule PD-L2, and this opens up the possibility of using these drugs in combination with other immunostimulatory compounds [26]. Therefore, the typical mechanism of anti-cancer drugs in general involves stimulating immune responses.

This mechanism plays an interesting role in regard to platinum anti-cancer drugs and the overall pathogenesis of tendinosis because immune cells may also be the source of tenogenic growth factors such as TGFβ ligands, which have been implicated in both fibrotic and regenerative tendon healing [27]. Therefore, the immunostimulatory nature of platinum-based anti-cancer drugs may actually be able to enhance tendon healing and regenerative properties, which could offset possible mechanisms of tendinosis from the drug as well. Hence, in the future, more research should be conducted to study the magnitude by which these anti-cancer drugs promote tendon healing through their immune-modulating effects and enhance tendinosis through collagen deterioration. Through this analysis, more conclusive evidence can be reached regarding whether these drugs definitively lead to tendon deterioration over time.

## Conclusions

Cancer patients who are prescribed platinum-based anti-cancer drugs often have a typical regimen of treatment that can last up to half a year. During this time, there are possible side effects, with one of them being acute tubular renal injury and electrolyte disturbances. These electrolyte imbalances can lead to a severely dehydrated and low volume state in these cancer patients, which can have drastic effects on both collagen production and stability. Collagen decomposition can lead to tendinosis and loss of function as tendons are predominantly made up of type 1 collagen. Therefore, there is a plausible effect of platinum-based cancer treatments leading to tendinosis in cancer patients who are on long-term chemotherapy regimens. However, the immunostimulatory nature of platinum-based anti-cancer drugs may actually be able to enhance tendon healing and regenerative properties, which could offset possible mechanisms of tendinosis from these drugs. Further research is needed on this subject, as tendinosis can severely affect cancer patients’ mobility and their range of motion and can be an essential factor when determining which antitumor regimen is best suited for a given patient at hand.
